# Emergence of *Escherichia coli* producing extended-spectrum AmpC β-lactamases (ESAC) in animals

**DOI:** 10.3389/fmicb.2014.00053

**Published:** 2014-02-14

**Authors:** Marisa Haenni, Pierre Châtre, Jean-Yves Madec

**Affiliations:** Agence Nationale de Sécurité Sanitaire, Unité Antibiorésistance et Virulence BactériennesLyon, France

**Keywords:** ESAC, *Escherichia coli*, bovine, animal, AmpC

## Abstract

In both humans and animals, the spread of Extended-Spectrum β-Lactamases (ESBL)/AmpC producers has become a major issue, particularly due to the plasmidic dissemination of most of these genes. Besides, over-expression of the chromosomal *ampC* gene was largely reported in human and animal *Enterobacteriaceae* and, more recently, modifications within the coding region of the *ampC* gene [encoding Extended-spectrum AmpC β-lactamases (ESACs)] were shown to be responsible for an hydrolysis spectrum expanded to oxyiminocephalosporins in humans. In this study, among 6765 cattle *E. coli* isolates, 28 (0.37%) isolates harboring a reduced susceptibility to cefepime (MICs ranging from 0.5 to 12 μg/ml) were investigated as presumptive ESACs producers. Highly conserved mutations in the promoter/attenuator region were identified at positions −88, −82, −42, −18, −1, and +58. Using sequencing and cloning experiments, amino acid substitutions of the AmpC beta-lactamase were characterized at positions 287 (mostly S287N, but also S287C), 292 (A292V) and 296 (H296P), similarly to data reported in humans. Interestingly, those cattle ESAC-producing *E. coli* isolates predominantly belonged to the Clonal Complex (CC) 23, thus mirroring what has been described in humans. The driving forces for the selection of ESACs in animals are unknown, and their prevalence needs to be further investigated in the different animal sectors. Considering the over-representation of ESAC-producing *E. coli* belonging to CC23 in both humans and animals, exchanges of ESAC producers between the two populations may have occurred as well. To our best knowledge, this study is the first report of ESACs in animals worldwide, which should be considered an emerging mechanism contributing to the resistance to extended-spectrum cephalosporins in the animal population.

## Introduction

*Escherichia coli* is both a commensal and an opportunistic pathogen of the digestive tract of mammals. *E. coli* is also responsible for many extra-intestinal infections (ExPEC), such as those of the urinary tract (Pitout, 2012). Concomitantly, the spread of resistances to extended-spectrum cephalosporins (ESCs) used in human and veterinary medicine causes major therapeutic challenges worldwide (Naseer and Sundsfjord, 2011). Indeed, in *E. coli*, the vast dissemination of Extended-Spectrum β-Lactamases (ESBLs) is a concern, in particular due to the broad success of the CTX-M enzymes. ESBL genes are mostly plasmid-mediated and this obviously facilitates their dissemination amongst bacteria (Carattoli, 2008). In addition, plasmidic class C beta-lactamases (AmpCs), such as the CMY-2 enzyme, also confer resistance to those last-generations antibiotics and disseminate efficiently in *Enterobacteriaceae*. In certain countries, a significant prevalence of CMY-2 producers in animals has been reported (Mulvey et al., 2009).

Numerous *Enterobacteriaceae* naturally produce a chromosome-encoded AmpC cephalosporinase. In *E. coli*, this enzyme is usually expressed at very low levels, as a result of a weak promoter and a transcriptional attenuator preceeding the *ampC* gene. Nevertheless, constitutive over-expression of the chromosomal *ampC* gene was largely reported in clinical isolates. This was attributed to specific spontaneous mutations in the promoter (which creates close homologies with the *E. coli* perfect consensus sequence) or the attenuator (which destabilizes the mRNA hairpin structure) of the *ampC* gene. Consequently, both mechanisms confer resistance to narrow-spectrum cephalosporins. In human medicine, oxyminocephalosporins, such as cefepime and cefpirome, remain usually active against over-expressed AmpC-producing *Enterobacteriaceae*, thanks to their rapid penetration through the outer membrane and poor degradation by AmpC beta-lactamases.

More recently, nucleotides substitutions within the coding region of the *ampC* gene were shown to be responsible for an expanded hydrolysis spectrum of AmpC enzymes to oxyiminocephalosporins (Mammeri et al., 2004a, 2006). Amino acid deletions or insertions (Doi et al., 2004; Mammeri et al., 2007) of the cephalosporinase also broaden the hydrolysis spectrum. Those so-called Extended-Spectrum AmpC β-lactamases (ESACs) were not only reported in *E. coli*, but also in other *Enterobacteriaceae*, such as *Enterobacter cloacae* (Crichlow et al., 1999; Barnaud et al., 2001; Vakulenko and Golemi, 2002), *Enterobacter aerogenes* (Barnaud et al., 2004), *Citrobacter freundii* (Ahmed and Shimamoto, 2008) or *Serratia marcescens* (Matsumura et al., 1998; Raimondi et al., 2001; Mammeri et al., 2004b; Hidri et al., 2005), and even in *Pseudomonas aeruginosa* and *Acinetobacter baumanii* (Rodriguez-Martinez et al., 2009, 2010). ESACs were considered an emerging mechanism of resistance to beta-lactams and their prevalence was estimated around 0.2%, almost identical to that of plasmidic cephalosporinases (Mammeri et al., 2008). Among the modifications of the *ampC* gene in ESAC-producing *E. coli*, certain nucleotides substitutions were more frequently identified, such as the S287N replacement. Other replacements were found in the coding sequence of plasmid-borne AmpCs (Kim et al., 2006), which also contribute to the extension of their hydrolysis spectrum. Finally, ESAC *E. coli* producers were predominantly found to belong to the Clonal Complex (CC) 23 (Cremet et al., 2010), and this raises the question on the selection scheme of those isolates.

To date, ESACs were reported in human isolates only. The purpose of this study was thus to detect and characterize the very first ESAC producers in animals worldwide.

## Materials and methods

### Bacterial isolates

A total of 6765 non-replicate *E. coli* isolates collected from cattle between February 2005 and December 2010 in France were included in this study. They were mostly recovered from fecal samples and from diseased animals (*n* = 6158), in particular from calves severely affected with gastro-enteritidis. Those isolates were recovered through the Resapath, the long-term surveillance network for antimicrobial resistance in pathogenic bacteria in France (www.resapath.anses.fr). An additional set of *E. coli* isolates (*n* = 607) collected from healthy French cattle (carriage) was also included. These latter isolates were recovered during a unique sampling program at slaughterhouse in 2006–2007. All isolates originated from various districts throughout the country and, when originating from the same district, came from different and widely distant farms. As the definition of AmpC over-producers may vary among studies (in particular with regard to resistance or not to expanded-spectrum cephalosporins in line with the strength of the *ampC* promoter), inclusion criteria for AmpC over-production were defined as follows, i.e., resistance to amoxicillin and amoxicillin-clavulanic acid, resistance to narrow-spectrum cephalosporins, reduced susceptibility to cefoxitin [diameter <22 mm, using the criteria of the Antibiogram Committee of the French Society for Microbiology (CA-SFM); (www.sfm-microbiologie.fr)] and resistance to ceftazidime (MIC ≥6 μg/ml) with a negative double-disk synergy test. For presumptive ESAC production, a reduced susceptibility to cefepime (MIC ≥0.5 μg/ml) was further added as an inclusion criterion.

### Antimicrobial susceptibility testing

Resistance to beta-lactams and non-beta-lactams was determined by the disc diffusion method according to the guidelines of the CA-SFM. *E. coli* ATCC 25922 was used as the quality control strain. The inhibitory effect of cloxacillin on AmpC production was observed on plates supplemented with 200 mg/L cloxacillin (AES Chemunex, Bruz, France). MICs to cefoxitin, ceftazidime, cefotaxime, and cefepime were determined by *E*-test (BioMérieux, Marcy l'Etoile, France) on all presumptive ESACs, and MICs to cefoxitin, ceftazidime, cefepime, and imipenem were also determined by *E*-test on the recombinant *E. coli* clones. As *E*-tests were not available for ceftiofur, MICs to ceftiofur were determined by broth dilution method on the recombinant *E. coli* clones.

### Phylogroup analysis and molecular typing of *E. coli* isolates

All *E. coli* isolates with reduced susceptibility to cefepime were assigned to a phylogenetic group (A, B1, B2, or D) using the PCR described by Clermont et al. (2000). Genetic relatedness of these isolates was determined by Pulse-Field Gel Electrophoresis (PFGE) after digestion with the *Bln*I restriction enzyme. Multi-Locus Sequence Typing was performed according to the scheme described on the *E. coli* MLST website (http://mlst.ucc.ie/mlst/dbs/Ecoli).

### Characterization and sequence analysis of the β-lactamase and ampC genes

The *bla*_SHV_, *bla*_TEM_, *bla*_OXA_, and *bla*_CTX−M_ genes were searched by PCR as previously described (Shibata et al., 2006; Dierikx et al., 2010). The plasmidic AmpC *bla*_CMY−2_ gene was sought using previously published primers (Mammeri et al., 2010). Chromosomal *ampC* promoter mutations were detected by PCR and sequencing of a 271 bp amplicon encompassing the −35 box, the −10 box and the attenuator (Caroff et al., 2000). The entire *ampC* gene of *E. coli* including its own promoter sequence was analyzed by PCR and sequencing using the Int-B2 and Int-H1 primers (Mammeri et al., 2006).

### Cloning of the *ampC* genes

The coding region (without the promoter) of *ampC* genes from *E. coli* with a reduced susceptibility to cefepime was amplified with primers Int-B1 and Int-HN, as previously described (Mammeri et al., 2006). These 1120 bp products were cloned into pCR BluntII TOPO (Invitrogen) and the recombinant plasmids were transformed into *E. coli* strain TOP10. Transformants were selected on plates containing kanamycin (50 μg/ml) and ceftazidime (1 μg/ml). The orientation of the insert was confirmed by PCR with the SP6 and T7 primers in order to only keep transformants with the *ampC* gene under the transcriptional control of the *lacZ* promoter.

## Results

### Prevalence of presumptive ESAC producers

Amongst the 6765 *E. coli* isolates studied, a total of 80 isolates (1.18%) met the criteria of AmpC over-production, of which 71 were clinical *E. coli* isolates (diarrheic calves) and 9 from healthy animals (carriage). Of them, 28 *E. coli* isolates harbored an additional reduced susceptibility to cefepime (MICs ranging from 0.5 to 12 μg/ml) and were investigated as presumptive ESACs producers (Table 1). MICs for ceftazidime for those 28 isolates ranged from 6 to 96 μg/ml, without a positive synergy test. Twenty-three presumptive ESACs producers (23/28, 82.1%) were from diseased cattle and 5 (5/28, 17.9%) were from healthy ones. None of the 28 *E. coli* had a plasmid-mediated *bla*_CMY_ gene, whereas 10 had a *bla*_TEM −1_ gene, 3 a *bla*_OXA−1_ gene, and 2 a *bla*_CTX−M−1_ gene. The 28 cattle were from different geographic origin in France, and *E. coli* were genetically unrelated (using PFGE, Table 1). However, most of them (18/28, 64.2%, Table 1) were of sequence type (ST) 88 belonging to the CC23. ST783 and ST1615 were also found four times each, respectively.

**Table 1:**
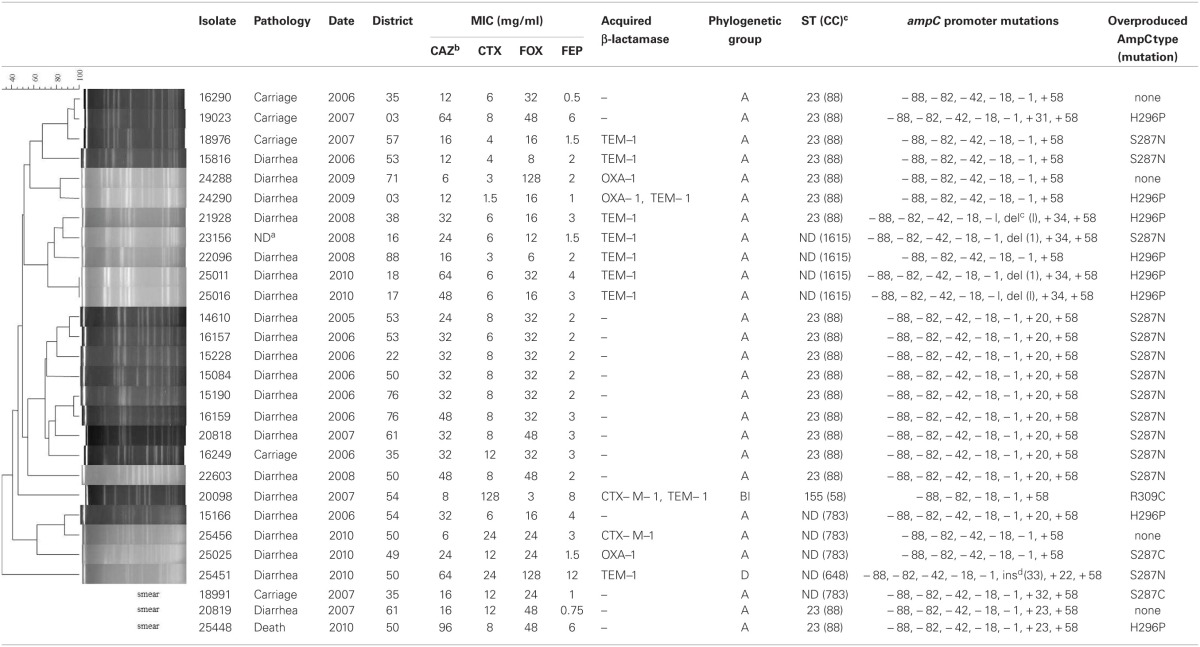
**Characteristics of the 28 *E. coli* strains presenting a reduced susceptibility to cefepime**.

^a^ND, not determined.

^b^CAZ, ceftazidime; CTX, cefotaxime; FOX, cefoxitin; FEP, cefepime.

^c^ST, sequence type; CC, clonal complex.

^d^del, deletion, ins, insertion.

### Mutations in the *ampC* promoter/attenuator and in the *ampC* gene

**Figure 1:**
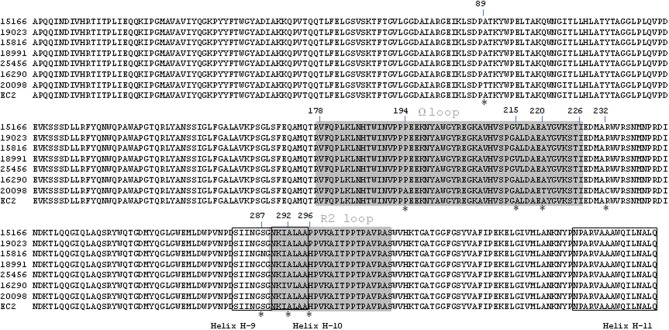
**Amino acids alignments of the ESAC β-lactamases** AmpC EC2 is a published narrow-spectrum cephalosporinase that was included as a reference strain. Point mutations are marked with a star. The two loops (Ω and R2) are highlighted in gray. Helix H-9, H-10, and H-11 are boxed.

Compared with the *E. coli* ATCC 25922, sequencing of the *ampC* promoter revealed highly conserved mutations at position −88, −82, −42, −18, −1, and +58. The number of nucleotides between the novel −35 and −10 sequences was 17 bp in all isolates. Mutations in the *ampC* gene attenuator (from +17 to +37) were frequent, particularly at position +20, but also at +23 or +34 (Table 1). Nucleotides deletion or insertion (at positions +1 or +33, respectively) were occasionally identified. Full sequencing of the *ampC* gene showed that amino acid substitutions of the AmpC beta-lactamase were mainly detected inside or near the H-10 helix (Figure 1) at positions 287 (*n* = 16; mostly S287N, but also S287C), 292 (*n* = 2, A292V) and 296 (*n* = 8; H296P), but also outside this hot spot at positions 89 (*n* = 1, A89T), 194 (*n* = 1, P194A), 215 (*n* = 4; A215V), 220 (*n* = 1; A220T), and 232 (*n* = 1, R232C) (Table 1 and Figure 1). The majority of the isolates presented one mutation in the coding sequence (*n* = 23), but three isolates presented two mutations and one isolate even presented four of them. Finally, one isolate which additionally harbored a CTX-M enzyme presented no mutation of the *ampC* gene compared the control.

### Characterization of the *ampC* recombinant *E. coli* clones

A subset of 7 *E. coli* isolates representative of the various amino acid substitutions found alone or in combination (A215V, A220T, S287N, S287C, A292V, H296P) and one control (15829, strain susceptible to cefepime) were chosen for cloning experiments (Table 2). PCR products of 1120 bp corresponding to the *ampC* coding sequence without its promoter were cloned into pCR BluntII TOPO. The seven recombinant plasmids were successfully transformed into *E. coli* strain TOP10, giving rise to clones TF-15166, TF-19023, TF-15816, TF-18991, TF-25456, TF-16290, TF-20098, and TF-15829 (susceptible control). In all recombinant plasmids, the insert was proved to be under the transcriptional control of the *lacZ* promoter. MICs of beta-lactams determined for the recombinant clones showed that the S287N substitution had a greater impact on resistance than S287C, H296P, and A292V (Table 2). TF-15166 and TF-19023 shared the same H296P mutation, but TF-15166 additionally harbored an A215V mutation that did not further increase the resistance to cefepime (MIC = 2 mg/L vs. MIC = 4 mg/L for TF-19023). The A215V had also weak impact on the susceptibility to cefepime when tested alone (TF-25456). Isolates 20098 displayed four different mutations (A89T, P194A, A220T, R232C), which do not increase resistance to cefepime (MIC = 0.19 mg/L) and were not found alone in the other tested isolates. TF-15829 (susceptible control) showed high MICs to ceftazidime (MIC = 24 mg/L) and cefoxitin (>256 mg/L) but a similar MIC value to cefepime (MIC = 0.125 mg/L) that TF-25456 or TF-20098. No mutation in the coding sequence of the *ampC* gene was found in TF-15829 compared to the reference strain.

**Table 2:** **Characteristics of the 7 *E. coli* transformants representative of the strains presenting a reduced susceptibility to cefepime**.

**Isolate**	**Date**	**MIC (mg/L) in transformants**					**Mutations in the *ampC* sequence**
		**CAZ^a^**	**FOX**	**FEP**	**IMI**	**XNL**	
**REDUCED SUSCEPTIBILITY TO CEFEPIME**							
TF-15166	2006	48	24	2	0.25	16	H296P, A215V
TF-19023	2007	>256	32	4	0.25	32	H296P
TF-15816	2006	>256	64	8	0.38	128	S287N
TF-18991	2007	96	32	1	0.25	64	S287C, A215V
TF-25456	2010	16	>256	0.125	0.25	32	A215V
TF-16290	2006	32	34	0.38	0.125	16	A292V
TF-20098	2007	8	48	0.19	0.5	16	A89T, P194A, A220T, R232C
**SUSCEPTIBLE CONTROLS**							
TF-15829	2006	24	>256	0.125	0.25	64	none
TOP 10	–	0.19	4	0.023	0.19	1	none

a CAZ, ceftazidime; FOX, cefoxitin; FEP, cefepime; IMI, imipenem; XNL, ceftiofur.

## Discussion

In this study, we report the first ESAC producers in animals worldwide. Contrary to humans (Barnaud et al., 2001; Mammeri et al., 2004b), ESACs were exclusively searched and found in the *E. coli* species. This results from the low proportion of animal infections due to other Gram negative bacteria expressing a chromosomal *ampC* gene, such as *S. marcescens*, *E. cloacae, E. aerogenes*, *A. baumanii*, or *P. aeruginosa* (Nordmann and Mammeri, 2007). Also, all ESAC-producing *E. coli* were from cattle as a result of our study design, and further investigations are surely needed to explore other animal sectors. The prevalence of ESAC-producing *E. coli* in cattle in France was investigated over a 6-year period of 2005–2010 and estimated at 0.37% (23/6158) in clinical isolates. Of note, this prevalence is similar to that found at a French hospital in the Paris suburb, where six ESAC producers were reported out of 2800 *E. coli* isolates (0.21%) (Mammeri et al., 2008). This prevalence is however four times higher than that found at another French hospital, where 41 AmpC-overproducing *E. coli* isolates accounted for 0.16% of all clinical *E. coli* isolates studied, of which only 11 produced ESACs (Cremet et al., 2010). However, in that study, inclusive criteria for ESAC producers included a MIC superior or equal to 64 mg/L of ceftazidime, which may have been a factor of underestimation.

Here, ESAC producers were mostly detected in diarrheic calves, which are also known as the main reservoir of ESBL genes in cattle (Madec et al., 2008, 2012; Valat et al., 2012). This may reflect the overall high antibiotic pressure with beta-lactams in infections of the enteric tract of young animals. However, a subset of 607 *E. coli* isolates from healthy cattle was also investigated, which revealed an even higher ESAC prevalence compared to diseased ones (5/607, 0.82%). This mirrors the situation in humans where ESAC producers were most likely found in *E. coli* belonging to the less pathogenic phylogroups A or B1 (Mammeri et al., 2006; Corvec et al., 2007). Moreover, the cattle ESAC-producing *E. coli* isolates predominantly belonged to the CC23, similarly to what has been reported in humans (Cremet et al., 2010). Further studies would be needed to understand why this evolution of the chromosomal *ampC* gene of *E. coli* seems restricted to the same genetic lineages in humans and animals despite different antimicrobial pressures and a rather host-specific distribution of the *E. coli* clones.

This study also provides an overview of 23 *ampC* promoter/attenuator sequences in cefoxitin-resistant *E. coli* from cattle. Contrary to humans, reports on mutations in the *ampC* promoter/attenuator regions of *E. coli* from animal sources are scarce (Brinas et al., 2002; Guillouzouic et al., 2009). The polymorphism at positions −88, −82, −42, −18, −1, +58 found in strong *ampC* promoters (Olsson et al., 1983; Guillouzouic et al., 2009) was highly conserved in our collection. In particular, this includes the −42 mutation shown to increase *ampC* gene expression (Nelson and Elisha, 1999; Caroff et al., 2000), and the association of the −42 and −18 changes, which creates two displaced new −35 and −10 boxes with an optimal 17-pb spacer region. The −18 mutation without the −42 mutation was rare (one isolate only) but had already been reported, in particular in *E. coli* isolates which do not over-express their chromosomal AmpC (Brinas et al., 2005). Other reported changes were not detected here, such as mutations −32, −11, or +6 (Caroff et al., 2000; Corvec et al., 2002; Mulvey et al., 2005; Tracz et al., 2007). However, these mutations might be more frequent in *E. coli* isolates of B2 or D phylogroups, which are not well represented here (Mammeri et al., 2008). The deletion of the entire attenuator was not observed either (Tracz et al., 2005).

In this study, all relevant amino acid replacements found in the *ampC* gene (namely S287N, S287C, A292V, and H296P) were single substitutions, confirming that cumulating substitutions in the catalytic site is not a preferential evolutionary scheme for AmpCs, contrary to class A beta-lactamases, as previously demonstrated (Le Turnier et al., 2009). A new A215V replacement was reported here in four *E. coli* isolates, either as a single or an associated mutation. This mutation, which occurs close to the DAEX motif in the Ω loop, has obviously little if no impact on the cefepime susceptibility. Indeed, in the corresponding native isolates, reduced susceptibility to cefepime may result from decreased permeability and/or oxacillinase production. Also, in the recombinant clones, the wild-type *ampC* gene of the cefepime susceptible control conferred the same MIC to cefepime than the A215V replacement. The main structural alteration found was the S287N replacement, which occurred in the H-9 helix close to the R2 loop. This mirrors the situation in humans where the S287N substitution has been shown to widely contribute to the ESAC phenotype (Mammeri et al., 2006). Similarly, other substitutions were also found, such as the S287C mutation, or both the H296P or the A292V mutations inside the R2 loop. Comparison of MICs of cefepime also confirmed that the S287N substitution has greater impact on resistance than the others (Mammeri et al., 2006).

Altogether this study is, to our best knowledge, the very first report of ESACs in animals. In humans, those enzymes have likely evolved from wild-type AmpCs by modifications in the vicinity of the R1 or R2 active sites. While the ESACs described here strongly resemble the human ones in both their genetic backgrounds and the responsible mutations, the driving forces for the selection of ESACs in animals are still unknown. In humans, the ESAC phenotype frequently occurs after cefepime therapy. On the contrary, different ESCs are used in animals, whose possible impact needs to be considered. In particular, ceftiofur is an ESC which is widely used in animals, and elevated MICs values to ceftiofur were found here in the recombinant *E. coli* clones. Hence, the use of veterinary cephalosporins in the selection of ESAC producers in animals might be a plausible hypothesis. Of note, most human and animal ESAC *E. coli* reported so far belong to the CC23—a well-adapted *E. coli* clone in the two hosts -, so that the transfer of ESAC producers from humans to animals may have contributed as well. Alternately, this may result from a favorable genetic background of the *ampC* genes in *E. coli* isolates of phylogroup A, irrespective of their human or animal origin (Mammeri AAC 2009). Even though data in humans report similar ranges of ESACs vs. plasmidic AmpCs (Mammeri et al., 2008), the true prevalence of ESACs in animals should be further investigated since this emerging mechanism surely contributes to the global burden of resistance to ESCs in animals.

### Conflict of interest statement

The authors declare that the research was conducted in the absence of any commercial or financial relationships that could be construed as a potential conflict of interest.

## References

[B1] Ahmed A. M. Shimamoto T. (2008). Emergence of a cefepime- and cefpirome-resistant *Citrobacter freundii* clinical isolate harbouring a novel chromosomally encoded AmpC beta-lactamase, CMY-37. Int. J. Antimicrob. Agents 32, 256–261 10.1016/j.ijantimicag.2008.04.019 18619820

[B2] Barnaud G. Benzerara Y. Gravisse J. Raskine L. Sanson-Le Pors M. J. Labia R. (2004). Selection during cefepime treatment of a new cephalosporinase variant with extended-spectrum resistance to cefepime in an *Enterobacter aerogenes* clinical isolate. Antimicrob. Agents Chemother. 48, 1040–1042 10.1128/AAC.48.3.1040-1042.2004 14982805 PMC353102

[B3] Barnaud G. Labia R. Raskine L. Sanson-Le Pors M. J. Philippon A. Arlet G. (2001). Extension of resistance to cefepime and cefpirome associated to a six amino acid deletion in the H-10 helix of the cephalosporinase of an *Enterobacter cloacae* clinical isolate. FEMS Microbiol. Lett. 195, 185–190 10.1111/j.1574-6968.2001.tb10519.x 11179650

[B4] Brinas L. Lantero M. De Diego I. Alvarez M. Zarazaga M. Torres C. (2005). Mechanisms of resistance to expanded-spectrum cephalosporins in *Escherichia coli* isolates recovered in a Spanish hospital. J. Antimicrob. Chemother. 56, 1107–1110 10.1093/jac/dki370 16239288

[B5] Brinas L. Zarazaga M. Saenz Y. Ruiz-Larrea F. Torres C. (2002). Beta-lactamases in ampicillin-resistant *Escherichia coli* isolates from foods, humans, and healthy animals. Antimicrob. Agents Chemother. 46, 3156–3163 10.1128/AAC.46.10.3156-3163.2002 12234838 PMC128764

[B6] Carattoli A. (2008). Animal reservoirs for extended spectrum beta-lactamase producers. Clin. Microbiol. Infect. 1, 117–123 10.1111/j.1469-0691.2007.01851.x 18154535

[B7] Caroff N. Espaze E. Gautreau D. Richet H. Reynaud A. (2000). Analysis of the effects of -42 and -32 ampC promoter mutations in clinical isolates of *Escherichia coli* hyperproducing ampC. J. Antimicrob. Chemother. 45, 783–788 10.1093/jac/45.6.783 10837430

[B8] Clermont O. Bonacorsi S. Bingen E. (2000). Rapid and simple determination of the *Escherichia coli* phylogenetic group. Appl. Environ. Microbiol. 66, 4555–4558 10.1128/AEM.66.10.4555-4558.2000 11010916 PMC92342

[B9] Corvec S. Caroff N. Espaze E. Marraillac J. Reynaud A. (2002). -11 Mutation in the ampC promoter increasing resistance to beta-lactams in a clinical *Escherichia coli* strain. Antimicrob. Agents Chemother. 46, 3265–3267 10.1128/AAC.46.10.3265-3267.2002 12234856 PMC128767

[B10] Corvec S. Prodhomme A. Giraudeau C. Dauvergne S. Reynaud A. Caroff N. (2007). Most *Escherichia coli* strains overproducing chromosomal AmpC beta-lactamase belong to phylogenetic group A. J. Antimicrob. Chemother. 60, 872–876 10.1093/jac/dkm284 17660264

[B11] Cremet L. Caroff N. Giraudeau C. Dauvergne S. Lepelletier D. Reynaud A. (2010). Occurrence of ST23 complex phylogroup A *Escherichia coli* isolates producing extended-spectrum AmpC beta-lactamase in a French hospital. Antimicrob. Agents Chemother. 54, 2216–2218 10.1128/AAC.01580-09 20145079 PMC2863611

[B12] Crichlow G. V. Kuzin A. P. Nukaga M. Mayama K. Sawai T. Knox J. R. (1999). Structure of the extended-spectrum class C beta-lactamase of *Enterobacter cloacae* GC1, a natural mutant with a tandem tripeptide insertion. Biochemistry 38, 10256–10261 10.1021/bi9908787 10441119

[B13] Dierikx C. Van Essen-Zandbergen A. Veldman K. Smith H. Mevius D. (2010). Increased detection of extended spectrum beta-lactamase producing *Salmonella enterica* and *Escherichia coli* isolates from poultry. Vet. Microbiol. 145, 273–278 10.1016/j.vetmic.2010.03.019 20395076

[B14] Doi Y. Wachino J.-I. Ishiguro M. Kurokawa H. Yamane K. Shibata N. (2004). Inhibitor-sensitive AmpC β-lactamase variant produced by an *Escherichia coli* clinical isolate resistant to oxyiminocephalosporins and cephamycins. Antimicrob. Agents Chemother. 48, 2652–2658 10.1128/aac.48.7.2652-2658.2004 15215122 PMC434168

[B15] Guillouzouic A. Caroff N. Dauvergne S. Lepelletier D. Perrin Guyomard A. Kempf I. (2009). MLST typing of *Escherichia coli* isolates overproducing AmpC beta-lactamase. J. Antimicrob. Chemother. 63, 1290–1292 10.1093/jac/dkp099 19307170

[B16] Hidri N. Barnaud G. Decre D. Cerceau C. Lalande V. Petit J. C. (2005). Resistance to ceftazidime is associated with a S220Y substitution in the omega loop of the AmpC beta-lactamase of a *Serratia marcescens* clinical isolate. J. Antimicrob. Chemother. 55, 496–499 10.1093/jac/dki025 15722393

[B17] Kim J. Y. Jung H. I. An Y. J. Lee J. H. Kim S. J. Jeong S. H. (2006). Structural basis for the extended substrate spectrum of CMY-10, a plasmid-encoded class C beta-lactamase. Mol. Microbiol. 60, 907–916 10.1111/j.1365-2958.2006.05146.x 16677302

[B18] Le Turnier S. Nordmann P. Eb F. Mammeri H. (2009). Potential evolution of hydrolysis spectrum for AmpC beta-lactamase of *Escherichia coli*. J. Antimicrob. Chemother. 63, 216–218 10.1093/jac/dkn443 18957396

[B19] Madec J. Y. Lazizzera C. Chatre P. Meunier D. Martin S. Lepage G. (2008). Prevalence of fecal carriage of acquired expanded-spectrum cephalosporin resistance in Enterobacteriaceae strains from cattle in France. J. Clin. Microbiol. 46, 1566–1567 10.1128/JCM.02299-07 18272707 PMC2292943

[B20] Madec J. Y. Poirel L. Saras E. Gourguechon A. Girlich D. Nordmann P. (2012). Non-ST131 *Escherichia coli* from cattle harbouring human-like *bla_CTX-M-15_*-carrying plasmids. J. Antimicrob. Chemother. 67, 578–581 10.1093/jac/dkr542 22210752

[B21] Mammeri H. Eb F. Berkani A. Nordmann P. (2008). Molecular characterization of *AmpC*-producing *Escherichia coli* clinical isolates recovered in a French hospital. J. Antimicrob. Chemother. 61, 498–503 10.1093/jac/dkm538 18250231

[B22] Mammeri H. Guillon H. Eb F. Nordmann P. (2010). Phenotypic and biochemical comparison of the carbapenem-hydrolyzing activities of five plasmid-borne *AmpC* beta-lactamases. Antimicrob. Agents Chemother. 54, 4556–4560 10.1128/AAC.01762-09 20733047 PMC2976168

[B23] Mammeri H. Nazic H. Naas T. Poirel L. Leotard S. Nordmann P. (2004a). AmpC beta-lactamase in an *Escherichia coli* clinical isolate confers resistance to expanded-spectrum cephalosporins. Antimicrob. Agents Chemother. 48, 4050–4053 10.1128/AAC.48.10.4050-4053.2004 15388478 PMC521871

[B24] Mammeri H. Poirel L. Bemer P. Drugeon H. Nordmann P. (2004b). Resistance to cefepime and cefpirome due to a 4-amino-acid deletion in the chromosome-encoded AmpC beta-lactamase of a *Serratia marcescens* clinical isolate. Antimicrob. Agents Chemother. 48, 716–720 10.1128/AAC.48.3.716-720.2004 14982755 PMC353140

[B25] Mammeri H. Poirel L. Fortineau N. Nordmann P. (2006). Naturally occurring extended-spectrum cephalosporinases in *Escherichia coli*. Antimicrob. Agents Chemother. 50, 2573–2576 10.1128/AAC.01633-05 16801449 PMC1489779

[B26] Mammeri H. Poirel L. Nordmann P. (2007). Extension of the hydrolysis spectrum of AmpC beta-lactamase of *Escherichia coli* due to amino acid insertion in the H-10 helix. J. Antimicrob. Chemother. 60, 490–494 10.1093/jac/dkm227 17586561

[B27] Matsumura N. Minami S. Mitsuhashi S. (1998). Sequences of homologous β-lactamases from clinical isolates of *Serratia marcescens* with different substrate specificities. Antimicrob. Agents Chemother. 42, 176–179 9449282 10.1128/aac.42.1.176 PMC105477

[B28] Mulvey M. R. Bryce E. Boyd D. A. Ofner-Agostini M. Land A. M. Simor A. E. (2005). Molecular characterization of cefoxitin-resistant *Escherichia coli* from Canadian hospitals. Antimicrob. Agents Chemother. 49, 358–365 10.1128/AAC.49.1.358-365.2005 15616316 PMC538860

[B29] Mulvey M. R. Susky E. McCracken M. Morck D. W. Read R. R. (2009). Similar cefoxitin-resistance plasmids circulating in *Escherichia coli* from human and animal sources. Vet. Microbiol. 134, 279–287 10.1016/j.vetmic.2008.08.018 18824313

[B30] Naseer U. Sundsfjord A. (2011). The CTX-M conundrum: dissemination of plasmids and *Escherichia coli* clones. Microb. Drug Resist. 17, 83–97 10.1089/mdr.2010.0132 21281129

[B31] Nelson E. C. Elisha B. G. (1999). Molecular basis of AmpC hyperproduction in clinical isolates of *Escherichia coli*. Antimicrob. Agents Chemother. 43, 957–959 10103209 10.1128/aac.43.4.957 PMC89235

[B32] Nordmann P. Mammeri H. (2007). Extended-spectrum cephalosporinases: structure, detection and epidemiology. Fut. Microbiol. 2, 297–307 10.2217/17460913.2.3.297 17661704

[B33] Olsson O. Bergstrom S. Lindberg F. P. Normark S. (1983). *ampC* beta-lactamase hyperproduction in *Escherichia coli*: natural ampicillin resistance generated by horizontal chromosomal DNA transfer from *Shigella*. Proc. Natl. Acad. Sci. U.S.A. 80, 7556–7560 10.1073/pnas.80.24.7556 6369321 PMC534379

[B34] Pitout J. D. (2012). Extraintestinal Pathogenic *Escherichia coli*: a combination of virulence with antibiotic resistance. Front. Microbiol. 3:9 10.3389/fmicb.2012.00009 22294983 PMC3261549

[B35] Raimondi A. Sisto F. Nikaido H. (2001). Mutation in *Serratia marcescens* AmpC beta-lactamase producing high-level resistance to ceftazidime and cefpirome. Antimicrob. Agents Chemother. 45, 2331–2339 10.1128/AAC.45.8.2331-2339.2001 11451693 PMC90650

[B36] Rodriguez-Martinez J. M. Nordmann P. Ronco E. Poirel L. (2010). Extended-spectrum cephalosporinase in *Acinetobacter baumannii*. Antimicrob. Agents Chemother. 54, 3484–3488 10.1128/AAC.00050-10 20547808 PMC2916328

[B37] Rodriguez-Martinez J. M. Poirel L. Nordmann P. (2009). Extended-spectrum cephalosporinases in *Pseudomonas aeruginosa*. Antimicrob. Agents Chemother. 53, 1766–1771 10.1128/AAC.01410-08 19258272 PMC2681535

[B38] Shibata N. Kurokawa H. Doi Y. Yagi T. Yamane K. Wachino J.-I. (2006). PCR classification of CTX-M-type beta-lactamase genes identified in clinically isolated gram-negative bacilli in Japan. Antimicrob. Agents Chemother. 50, 791–795 10.1128/aac.50.2.791-795.2006 16436748 PMC1366867

[B39] Tracz D. M. Boyd D. A. Bryden L. Hizon R. Giercke S. Van Caeseele P. (2005). Increase in *ampC* promoter strength due to mutations and deletion of the attenuator in a clinical isolate of cefoxitin-resistant *Escherichia coli* as determined by RT-PCR. J. Antimicrob. Chemother. 55, 768–772 10.1093/jac/dki074 15761065

[B40] Tracz D. M. Boyd D. A. Hizon R. Bryce E. McGeer A. Ofner-Agostini M. (2007). *ampC* gene expression in promoter mutants of cefoxitin-resistant *Escherichia coli* clinical isolates. FEMS Microbiol. Lett. 270, 265–271 10.1111/j.1574-6968.2007.00672.x 17326753

[B41] Vakulenko S. Golemi D. (2002). Mutant TEM beta-lactamase producing resistance to ceftazidime, ampicillins, and beta-lactamase inhibitors. Antimicrob. Agents Chemother. 46, 646–653 10.1128/AAC.46.3.646-653.2002 11850243 PMC127477

[B42] Valat C. Auvray F. Forest K. Metayer V. Gay E. Peytavin De Garam C. (2012). Phylogenetic grouping and virulence potential of extended-spectrum-beta-lactamase-producing *Escherichia coli* strains in cattle. Appl. Environ. Microbiol. 78, 4677–4682 10.1128/AEM.00351-12 22522692 PMC3370483

